# Expert Recommendations on Monkeypox (MPX) in Pregnancy, Postpartum and Lactating Women

**DOI:** 10.1055/s-0042-1759635

**Published:** 2022-12-29

**Authors:** Rosiane Mattar, Antonio Rodrigues Braga Neto, Adriana Gomes Luz, Alan Hatanaka, Alberto Zaconeta, Cristina Aparecida Falbo Guazzelli, Evelyn Traina, Fernanda Spadotto Baptista, Gabriel Osanan, Geraldo Duarte, Jose Geraldo Lopes Ramos, Maria Lucia Oppermann, Rossana Pulcineli Vieira Francisco, Sigrid Maria Loureiro de Queiroz Cardoso, Silvana Maria Quintana, Sue Yazaki Sun, Vera Therezinha Medeiros Borges

**Affiliations:** 1 Escola Paulista de Medicina, Universidade Federal de São Paulo, São Paulo, SP, Brazil; 2 Universidade Federal do Rio de Janeiro, Rio de Janeiro, RJ, Brazil; 3 Universidade Federal Fluminense, Niterói, RJ, Brazil; 4 Universidade Estadual de Campinas, Campinas, SP, Brazil; 5 Universidade de Brasília, Brasília, DF, Brazil; 6 Faculdade de Medicina, Universidade de São Paulo, São Paulo, SP, Brazil; 7 Universidade Federal de Minas Gerais, Belo Horizonte, MG, Brazil; 8 Faculdade de Medicina, Universidade de São Paulo, Ribeirão Preto, SP, Brazil; 9 Universidade Federal do Rio Grande do Sul, Porto Alegre, RS, Brazil; 10 Universidade Federal do Amazonas, Manaus, AM, Brazil; 11 Faculdade de Medicina, Universidade Estadual Paulista, Botucatu, SP, Brazil

## The Monkeypox Disease

In 2020, Brazil and the whole world faced the COVID-19 pandemic, which caused a high number of deaths. This disease was particularly severe for pregnant and postpartum women and determined a significant increase in the Maternal Death Ratio (MMR). To face the disease and assist health professionals in the qualification of the best care to the maternal-fetal binomial, the Ministry of Health and Febrasgo developed a working group formed by professors and researchers from several universities who worked to establish recommendations for the care of pregnant women and puerperal women by the time of the COVID-19 pandemic.

In 2022, while we are still experiencing the COVID-19 pandemic, we are surprised by another disease caused by a virus that has been alarming the population and worrying public health authorities and gynecology and obstetrics societies in Brazil and worldwide.

It is the infection that is caused by monkeypox virus (MPXV), which is still a not well-known disease, with many of its characteristics not well determined. The knowledge of this disease is fundamental for health professionals working in Obstetrics to plan forms of prevention, as well as the establishment of the diagnosis and treatment of the monkeypox (MPX) disease, preserving the health of the maternal-perinatal binomial. For this reason, the Brazilian Ministry of Health requested the same working group that acted diligently by the time of COVID-19 to establish recommendations for facing MPX, to provide adequate care for pregnant women and puerperal women. These recommendations, based on the knowledge that exists so far, are what guide these orientations and may change depending on new findings that may be presented over time.

The MPXV was named after being identified in laboratory monkeys in 1958. The first case of this virus in humans was recorded in 1970 in a child in Congo and since then has become an endemic disease in West and Central Africa. 1 In 2003, the first cases were registered outside the African continent, in the United States, 2 3 4 but that was contained through hygienic measures and stock vaccines. In 2017, there was a major outbreak started in Nigeria and spread to some African countries. In early May 2022, another outbreak of MPXV was identified, this time in several countries outside the African continent, with fast dissemination of cases. As a result, on May 21, 2022, the World Health Organization (WHO) declared the existence of an emerging global outbreak of MPXV infection, and on July 23 has determined that this outbreak constituted a Public Health emergency of international concern.

Pregnant women present clinically with similar characteristics to nonpregnant women, but may evolve with greater severity, being therefore considered a risk group. In addition to maternal clinical repercussions, there are also concerns specific to the pregnancy period, such as fetal vitality, the possibility of vertical transmission and perinatal outcome. It has been verified that MPXV infection can lead to adverse results in pregnancy, such as fetal death and spontaneous abortion. 5 6 A recent publication on the evolution of pregnancy in 4 MPXV-infected women showed spontaneous 1 ^st^ trimester abortion in 2 pregnant women, without testing of the conception products; an intrauterine death in the 2 ^nd^ trimester, with clinical, histological and laboratory evidence of intrauterine fetal infection evidencing the very probable vertical transmission of the disease, and a pregnant woman with MPXV infection that evolved with full-term delivery of healthy conceptus. 7

Close and prolonged skin-to-skin contact, including during sexual activity, seems to be the main means of transmission of MPXV. There are suspicions of transmission of this virus by droplets and aerosols. There is also transmission through biting of rodent animals or even the ingestion of those animals. In addition, contagion by phositis, especially used clothing, can transmit the disease. The quick identification and isolation of affected individuals is fundamental to prevent the spreading of the disease. 8

Transmission of MPXV occurs in the phase of active skin lesions and only ends when they heal completely, which usually requires isolation of 21 to 28 days. 7 9 10 There are doubts as to whether the contagion could be prior to the phase of skin lesions, since viral DNA has already been identified in the blood and respiratory system of patients prior to the lesions. 5 7 Sexual transmission has been discussed not only by contact, but also because the virus has been identified in seminal material. 5 11

Patients with MPXV should be isolated in a separate area of their home or in hospital services, especially if they present extensive lesions and/or respiratory symptoms. Skin lesions should be covered (for example, with the use of long sleeves and trousers) to minimize the risk of contact. Everyone should wear a face mask in the presence of an infected person.

Sexual abstinence is also recommended in the phase of unhealed lesions and condom use for any form of sexual act (anal, oral, or vaginal) in the 12 weeks following the healing of the lesions. 7

Most patients with MPXV will have mild disease and can be cared for at home, where they should remain isolated. Standard cleaning and disinfection procedures should be performed, taking care of clothes and used objects.

The diagnosis of infection can be made by anamnesis and clinical findings, with epidemiological suspicion. The incubation period is, on average, 6 to 13 days, and can be from 5 to 21 days. Next to this, a prodromal period occurs, when fever, sweating, headache, myalgia, fatigue and lymphadenomegaly, which is quite characteristic of the disease, are manifested. About 1 to 3 days later, the rash, which usually affects the face, genitals, and extremities, and has a centrifugal character, appears.

The lesion evolves from macules to papules, vesicles, pustules and, later, crusts. 3 In general, they are well circumscribed and deep, and develop umbilication. They can also be uniform or at different stages of evolution. Lesions are often painful until healing (which happens normally when they are itching).

The severity criteria consider the number of lesions: Mild (< 25 skin lesions), Moderate (25–99), Severe (100–250) and very severe (> 250). Another severity criterium is when one of these signs is present: fever for > 7 days, cervical lymph node, persistent vomiting, dehydration, retrocular pain, respiratory failure, mental confusion, hepatomegaly, and sepsis.

Confirmatory laboratory diagnosis of MPXV can be performed by real-time polymerase chain reaction (qPCR). 12 Sample collection should preferably be performed with swab of ulcerated lesions. If there are only vesicles, the sample can be obtained by means of fine needle puncture, with extreme caution to avoid accidents that allow contagion. If there are only crusts, the material can be obtained by swab or by collection of small fragments. These samples should be stored in dry sterile vials without any preserving liquid. In individuals in whom MPXV is suspected, without any clinical suspicion, oral swab should be tested and vaginal or anal swab may be considered. 13

Blood samples are oriented for differential diagnosis and/or concomitance of other diseases that could cause lesions, such as syphilis, acquired immunodeficiency syndrome, and herpetic infection. 12 13

Although the disease is most of the time self-limited and with spontaneous cure, in some cases there may be a need for specific drug treatment. Most of the time, there is only indication of symptomatic treatment for fever and pain with dipyrone, acetaminophen, or even opiate derivatives in the most severe pain conditions. In cases with more important lesions, the use of antibiotics may be indicated to prevent secondary bacterial infection: systemic amoxicillin and ocular chloramphenicol.

There are some patients who have worsening of the condition, and in these circumstances, antivirals are indicated. Antiviral drugs: Tecovirimat (TPOXX), cidofovir (Vistide) and brincidofovir (Tembexa) have been considered. About 5% of patients with MPXV require antivirals. 11 14 There are still no well-established protocols for their use in pregnancy.

Immunoglobulin (VIG), a mixture of purified blood antibodies of individuals immunized with the smallpox vaccine, has already been used for the prevention/treatment of MPXV. There is no evidence on its effectiveness; however, it has already been considered as prophylaxis in exposed individuals with severe immunodeficiency as a prevention/treatment of MPXV.

There are two vaccines developed to fight human smallpox, which are capable of inducing protective antibodies against MPXV. However, they are not available in Brazil and there are not enough doses for mass vaccination.

The first is ACAM2000, with live vaccinia attenuated but replicating virus, applied in a single dose and with immune response 4 weeks after application. Because it is a live virus vaccine, it is contraindicated for individuals with immunodeficiency and pregnant women.

The second vaccine is Modified Ankara (MVA-BN), produced by Bavarian Nordic, with live, attenuated, nonreplicating virus 15 is sold in Europe as Imvamune or Imvanex and in the United States as Jynneos. It has efficacy of 85%. 16 It can be used in immunosuppressed patients. It is applied in two doses with an interval of 4 weeks, and its protection begins 2 weeks after the second dose.

## Monkeypox in Pregnant, Postpartum, and Lactating Women

There are few reports on MPXV during pregnancy. 6 It is known that the virus can cross the placenta and reach the fetus. Thus, as in other viral infections, it may increase the risk of abortion, fetal death, prematurity, and other fetal complications. There is still no way to quantify these risks. Therefore, the care with the pregnant woman and the fetus should be intense in the face of suspicion or confirmation of the infection.

The WHO recognizes maternal-fetal transmission through the transplacental passage, originating the congenital disease, and/or transmission through intimate contact, during and after delivery. In postexposure asymptomatic pregnant women, if MPXV is undetectable, monitoring can be suspended. If MPXV is detectable, home isolation should be maintained for a minimum of 21 days. Self-monitoring of body temperature and skin lesions and teleservice monitoring by the health professionals should be orientated.

In pregnant women with signs or symptoms of MPXV but with negative qPCR, isolation and self-monitoring of temperature and skin lesions should be indicated. Other potential causes should be ruled out, and retesting the patient is indicated if symptoms persist. If the MPXV test is positive, hospitalization is indicated in moderate and severe cases. There are still insufficient data on the use of vaccines in pregnant or lactating women and none of the vaccines are approved in pregnancy. Animal studies did not find adverse fetal effects, and a study with 300 pregnant women did not show an increase in adverse outcomes. 17 It is still unknown whether vaccines are excreted in breast milk. Vaccines with replicant viruses are contraindicated in pregnancy and infants. 18 19 20 The MVA-BN has been considered safe during breastfeeding. 1 Thus, any woman who is breastfeeding, with substantial exposure to the virus, should be vaccinated after considering the risks of MPXV infection for her and her child.

It will usually only be necessary to use symptomatic for the treatment of MPVX during pregnancy. The use of antivirals is not approved. There are no studies of the antiviral drugs in humans. In animals, Tecovirimat did not induce teratogenicity, and Cidofovir and Brincidofovir were classified as FDA class C because they caused changes in the morphology of the animals undergoing the study. Although little is known about VIG during pregnancy, other immunoglobulins have already been used in pregnancy and have been shown to be safe. Until now, this type of treatment has not been indicated during pregnancy. 18

In the presence of acute infection, obstetric ultrasound (US) is recommended during the 1 ^st^ trimester to evaluate the viability of pregnancy. In moderate and severe cases during the 2 ^nd^ trimester, obstetric US is suggested to evaluate biometrics and fetal morphology as well as to quantify the amniotic fluid index. During the 3 ^rd^ trimester, when available, fetal biophysical profile and fetal Doppler flowmetry should be done to assess the well-being of the conceptus. After the 26 ^th^ week, cardiotocography is recommended in cases of infection considered moderate and severe. 19

After the maternal cure, fetal risks are low, but obstetric US is recommended every 4 weeks for evaluation of fetal growth and well-being. 10 Normally, there is no indication to anticipate delivery. 7 In severe cases or fetal impairment, we should consider delivery, evaluating gestational age and fetal weight. In cases in which preterm delivery is indicated, magnesium sulfate and corticosteroids should be used, according to obstetric indication.

During the delivery, the presence of a healthy companion with the use of personal protective equipment should be ensured, which should be maintained throughout the hospital stay. The delivery should follow obstetrics indications, and there is no reason to indicate cesarean section because of the infection. If the patient presents genital lesions, because of a higher risk of neonatal infection during delivery, c-section will be indicated. 9

Timely clamping of the umbilical cord is recommended, although skin-to-skin contact between mother and newborn (NB) should be avoided. Immediate macroscopic examination of the NB should be taken and, when available, a swab of throat and any skin lesions. 9 It is recommended that the newborn be sanitized by bathing immediately after delivery. It is up to the doctor to inform the risks of infection and need to keep mother and child in separate rooms during the isolation phase. If this is not possible, strict precautions should be followed during contact: the NB should be fully clothed or wrapped in sheets, just as the mother should wear gloves and surgical mask well-adjusted to the face. Direct breastfeeding should be postponed, but support should be offered for the woman to maintain milk production and allow relactation later. Milked breast milk should be discarded. Precautions should be maintained until isolation criteria are met. If the NB is tested positive, the isolation can be cleared. Action of antiviral drugs and vaccine immunoglobulin are little known in milk production. 20 The discharge should be adjusted considering the isolation time and the ability to follow to the recommendations to avoid the contagion of the NB.

## Recommendations for Pregnant, Postpartum, and Lactating Women

- Use of masks, especially in environments with individuals potentially infected with the virus.
- Stay away from people who have suspected symptoms such as fever and mucosal skin lesions.
- Use condoms in all types of sexual intercourse (oral, vaginal, anal) since transmission through intimate contact has been the most frequent.
- Be alert if your sexual partnership presents any lesion in the genital area.
- Seek for medical attention if you have a suspicious symptom, so that a clinical and laboratory diagnosis can be established.

## Recommendations for healthcare professionals

- Pregnant women should be at home isolated with constant follow-up by the care team in case of mild illness.
- Cases of greater severity should be followed-up in hospital.
- There is still no specific treatment protocol with antivirals in the pregnancy-puerperal cycle.
- Monitoring fetal vitality should be carefully observed in patients with moderate or severe disease, because of the higher fetal morbidity and mortality in these cases.
- The delivery has obstetrics indications and cesarean section as a routine is not indicated.
- Breastfeeding should be postponed during the isolation period, offering specific support that allows for further relactation.
